# Significance in scale space for Hi-C data

**DOI:** 10.1093/bioinformatics/btaf026

**Published:** 2025-02-27

**Authors:** Rui Liu, Zhengwu Zhang, Hyejung Won, J S Marron

**Affiliations:** Department of Statistics and Operations Research, University of North Carolina at Chapel Hill, Chapel Hill, NC 27599, United States; Department of Statistics and Operations Research, University of North Carolina at Chapel Hill, Chapel Hill, NC 27599, United States; Department of Genetics, University of North Carolina at Chapel Hill, Chapel Hill, NC 27599, United States; Department of Statistics and Operations Research, University of North Carolina at Chapel Hill, Chapel Hill, NC 27599, United States

## Abstract

**Motivation:**

Hi-C technology has been developed to profile genome-wide chromosome conformation. So far Hi-C data have been generated from a large compendium of different cell types and different tissue types. Among different chromatin conformation units, chromatin loops were found to play a key role in gene regulation across different cell types. While many different loop calling algorithms have been developed, most loop callers identified shared loops as opposed to cell-type-specific loops.

**Results:**

We propose SSSHiC, a new loop calling algorithm based on significance in scale space, which can be used to understand data at different levels of resolution. By applying SSSHiC to neuronal and glial Hi-C data, we detected more loops that are potentially engaged in cell-type-specific gene regulation. Compared with other loop callers, such as Mustache, these loops were more frequently anchored to gene promoters of cellular marker genes and had better APA scores. Therefore, our results suggest that SSSHiC can effectively capture loops that contain more gene regulatory information.

**Availability and implementation:**

The Hi-C data used in this study can be accessed through the PsychENCODE Knowledge Portal at https://www.synapse.org/#! Synapse: syn21760712. The code utilized for Curvature SSS cited in this study is available at https://github.com/jsmarron/MarronMatlabSoftware/blob/master/Matlab9/Matlab9Combined.zip. All custom code used in this research can be found in the GitHub repository: https://github.com/jerryliu01998/HiC. The code has also been submitted to Code Ocean with the doi: 10.24433/CO.1912913.v1.

## 1 Introduction

In the rapidly advancing field of genomics, the comprehensive understanding of genomic organization has been greatly enhanced by the advent of Hi-C technology (Fraser *et al.* 2015, Rocha *et al.* 2015, Pal *et al.* 2019, Lafontaine *et al.* 2021). This sophisticated method has reshaped our comprehension of the three-dimensional architecture of genomes, extending our insight beyond the mere linear sequence of DNA (Lieberman-Aiden *et al.* 2009, Dixon *et al.* 2012). Hi-C, a technique developed in the late 2000s, is based on chromosome conformation capture (3C) techniques (Lieberman-Aiden *et al.* 2009, Belton *et al.* 2012, Rao *et al.* 2014). It involves crosslinking DNA to preserve local three-dimensional interactions, followed by enzymatic digestion, re-ligation, and high-throughput sequencing (Pal *et al.* 2019, Lafontaine *et al.* 2021). This methodology enables the identification of physical contacts between distant genomic regions (Bonev and Cavalli 2016, Marchal *et al.* 2019, Zheng and Xie 2019).

Chromatin loops, identified as pairs of genomic locations showing a contact frequency significantly above what would be anticipated from random collisions, were first detected through extensive sequencing of Hi-C data from mass cell samples (Rao *et al.* 2014). These loops, pivotal for bringing distant genomic regions into proximity, improve crucial interactions between enhancers and promoters, thus governing the transcriptional landscape and cell fate decisions (Mora *et al.* 2016, Lu *et al.* 2020, Hsieh *et al.* 2022, Goel *et al.* 2023). However, the task of precisely identifying these loops is significantly complicated by the inherent sparsity present in Hi-C data. This sparsity challenges the sensitivity and specificity of loop detection algorithms, requiring sophisticated computational approaches that can detect the biologically meaningful signals from the large, sparse dataset (Yu *et al.* 2021, Li *et al.* 2022).

Detecting cell-type-specific loops is crucial in this field because it allows researchers to understand how the three-dimensional organization of the genome influences gene expression and regulatory mechanisms in a cell-type-specific manner (Fernandez *et al.* 2020, Kim *et al.* 2020). Loop callers such as HiCCUPS (Rao *et al.* 2014), SIP (Rowley *et al.* 2020), and Mustache Roayaei (Ardakany *et al.* 2020) have successfully identified chromatin loops from the Hi-C data. However, the loops detected by these algorithms often represent structural loops that do not overlap with gene promoters (Nicoletti *et al.* 2018). Furthermore, due to the nature of structural loops, they are frequently not cell type specific, making it difficult to identify enhancer–promoter landscapes that vary across cell types.

In this paper, we develop SSSHiC, a new Hi-C loop caller, which addresses this critical issue. SSSHiC is based on Significance in Scale Space Chaudhuri and Steven Marron (2000) (Godtliebsen *et al.* 2004, 2002) utilizing its statistical inference capabilities with a focus on curvature analysis. Unlike the original method, SSSHiC excludes slope analysis and visualization features, adapting only the peak curvature component for Hi-C loop detection. Scale space analysis (Lindeberg 1994, 2009) is predicated on the idea that data can be understood more comprehensively when analyzed at multiple scales or resolutions. This concept, originating from the fields of signal processing and computer vision (Witkin 1984, Lindeberg 1994), has been adapted to statistical analysis to address the challenges posed by data exhibiting features at various scales. The method involves systematically smoothing data over a continuum of scales and analyzing these transformations to identify statistically significant features and patterns (Chaudhuri and Marron 1999, Chaudhuri and Sarkar 1999). Methodologically, scale-space analysis in statistics involves the application of various techniques such as kernel smoothing and Gaussian filters (Babaud *et al.* 1986, Lindeberg 1994, Sporring *et al.* 2013). These techniques allow for the gradual blurring or smoothing of data, revealing structures and features at different levels of resolution. The choice of method and scale parameters depends on the nature of the data and the specific objectives of the analysis (Chaudhuri and Sarkar 1999).

SSSHiCis applied to Hi-C datasets obtained from two brain cell types (Neuron and Glia). Below, we demonstrate the utility of SSSHiCin identification of cell-type-specific loops.

## 2 Materials and methods

### 2.1 Input data

We used Hi-C data from neuron and glia which are two major cell classes in the human brain (Hu *et al.* 2021). In SSSHiC, we binned the Hi-C matrix at 10-kb resolution (other resolutions are possible as well) and identified chromatin loops.

Let $\tilde{X}=\{{\tilde{x}}_{ij}\}$ be a bin count matrix for each chromosome for a given cell type, where ${\tilde{x}}_{ij}$ is the number of contacts between bins *i* and *j*. For computational tractability, we use sliding windows (d×d matrices) whose step sizes are d/2. Hence each window is overlapped with its adjacent window through a sub-matrix of size d/2×d/2. Choice if *d* represents a trade-off between window size and the number of windows, with several reasonable potential choices. In this paper, we always use d=1000.

### 2.2 Preprocessing for Hi-C

Let ${\tilde{X}}_{k}=\{{\tilde{x}}_{ij}^{k},1\leq i,j\leq d\}$ denote the *k*th step sliding window. First, we take a log transformation of each count in the Hi-C matrix, i.e. let $x_{ij}^{k}=\log({\tilde{x}}_{ij}^{k}+1)$ (plus 1 since some elements of the matrix are 0) with resulting data matrix $X_{k}$.

We work with two cell types, neurons whose *k*th step window is denoted as $X_{k}^{N}$ and glia as $X_{k}^{G}$. These represent the same location in the chromosome but from different cell types. Figure 3A shows the distribution of the $x_{ij}^{k}$ for neurons (red) and glia (blue), as bars indicating zero counts and the kernel density estimates of the nonzero counts. Since the scales and distributions of different cell types are different, rescaling the data is necessary. To adjust for differences in read depth and library quality, let $m_{k}^{N}$ and $m_{k}^{G}$ be the median values of the nonzero entries of $X_{k}^{N}$ and $X_{k}^{G}$ shown as vertical lines, respectively. For the distribution with larger median value (neuron for this window), the difference between medians is subtracted from each nonzero entry. Any negative element of the resulting matrix is set to 0. Thus, the neuron and glia Hi-C matrices will have the same median value of their nonzero elements as shown in Figure 3B. This process is called *median matching*.

**Figure 1.**
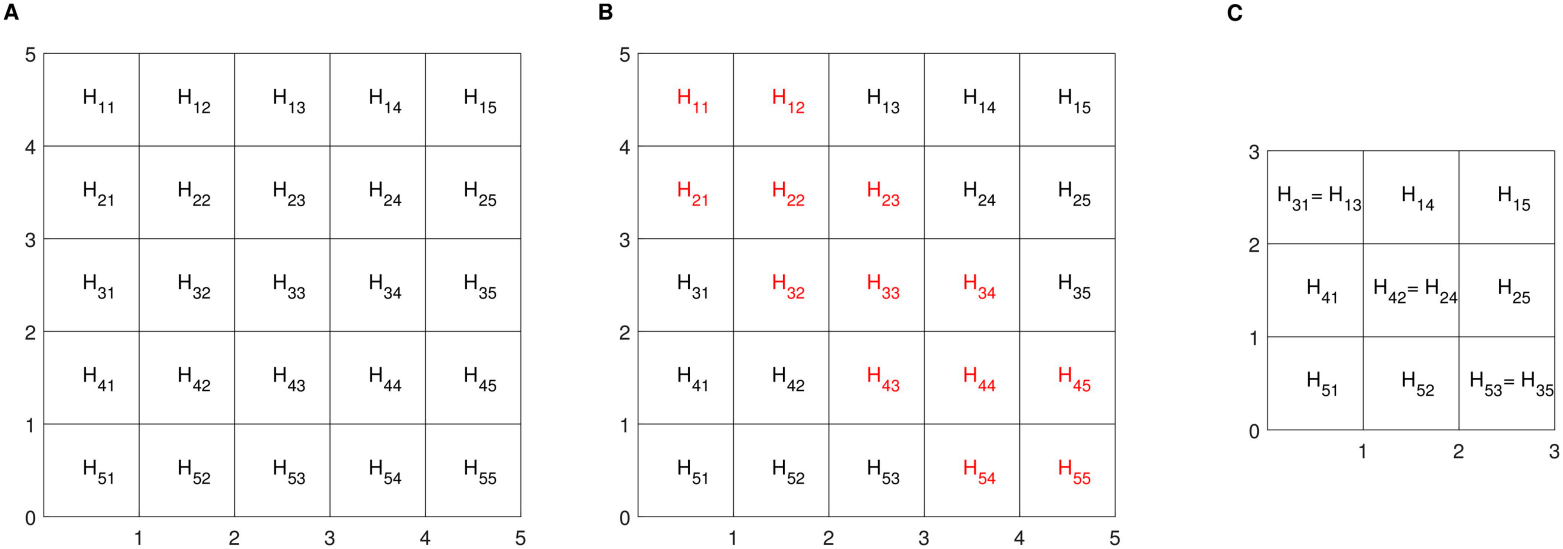
A toy example of a 5×5 symmetric data matrix in the left panel. Diagonal and off-diagonal lines entries to be removed, marked in the middle panel and the resulting (5−1−1)×(5−1−1) matrix in the right panel.

Because the large diagonal entries of each matrix $\left\{x_{ii}\right\}$ strongly impact our analysis, we remove them. Furthermore, we increase the detection accuracy of long-range interactions by removing a band of diagonal lines, indexed by offsets 1,…,c, of off-diagonal entries as well. The left panel of Figure 1 shows a toy example data window. The diagonal and off-diagonal lines to be removed are highlighted in red. The resulting (d−c−1)×(d−c−1) matrix is shown in the right panel.

### 2.3 Significance in scale space for loop definition

In order to detect loops in the Hi-C map, SSSHiC uses the curvature version of Significance in Scale Space (SSS) (Godtliebsen *et al.* 2004), which is a technology for finding which features in a noisy image are strong enough to be distinguished from background noise, in the sense of statistical significance. Note that the Hi-C matrix is quite noisy, and it is not clear which loops are really there. SSS uses Gaussian window smooths to reduce the noise in an image.

The statistical model is

(1) $$Y_{i,j}=s(i,j)+\epsilon_{i,j},$$

where i,j=1,…,d index pixel locations, $\epsilon_{i,j}$ is the independent random noise, and *s* represents a relatively smooth underlying true signal. The signal *s* can be estimated using a Gaussian window smoother

(2) $${\hat{s}}_{h}(i,j)=\sum_{k=1}^{d}\sum_{l=1}^{d}Y_{k,l}K_{h}(i-k,j-l),$$

where $K_{h}$ is a Gaussian probability density with mean 0 and covariance matrix $h^{2}$ times the identity matrix. An important point is that our methodology goes far beyond merely smoothing for noise reduction. In particular, we also do fundamental statistical inference by assigning statistical significance to peaks. In SSS software, we can use dynamic graphics (i.e. movies) based on scale as a time parameter to visualize the persistance of features across scales.

Peaks in surfaces are characterized by curvature which is quantified by second derivatives. Simple second derivatives reflect curvatures in the function f(x,y) in the direction of the coordinate axes. Curvatures in arbitrary directions are captured by the Hessian matrix,

$$H(f)=\left(\begin{array}{cc} \frac{\partial^{2}f}{\partial x^{2}} & \frac{\partial^{2}f}{\partial x\partial y}\\ \frac{\partial^{2}f}{\partial y\partial x} & \frac{\partial^{2}f}{\partial y^{2}} \end{array}\right).$$

The eigenvalues of this matrix, denoted by $\lambda_{+}$ and $\lambda_{-}$, provide insight into the nature of the surface curvature at a given point. Curvature is quantified in a rotation invariant way by consideration of the Eigenvalues of the Hessian. If all of the eigenvalues are negative (positive), it is said to be a negative-definite (positive-definite) matrix, indicating concavity. Since peaks are points of maximal concavity, their statistical significance is quantified by significance of the eigenvalues of the Hessian matrix.

Statistical inference for other geometric features can similarly be established using eigenvalues of the Hessian matrix as

**Table:** 

Feature	Characterization
Hole	${\hat{\lambda }}_{+},{\hat{\lambda }}_{-}>{\hat{q}}_{T}$
Long valley	${\hat{\lambda }}_{+}>{\hat{q}}_{T},|{\hat{\lambda }}_{-}|<{\hat{q}}_{T}$
Saddle point	${\hat{\lambda }}_{+}>{\hat{q}}_{T},{\hat{\lambda }}_{-}<-{\hat{q}}_{T}$
Long ridge	$|{\hat{\lambda }}_{+}|<{\hat{q}}_{T},{\hat{\lambda }}_{-}<-{\hat{q}}_{T}$
Peak	${\hat{\lambda }}_{+},{\hat{\lambda }}_{-}<-{\hat{q}}_{T}$

For example, the strength of each potential loop can be quantified through the parameter $T_{i,j}=\max\{\lambda_{+},\lambda_{-}\}$, and the null hypothesis is $H_{0}:T_{i,j}=0$ and the alternative hypothesis is $H_{a}:T_{i,j}<0$. As detailed by Godtliebsen *et al.* (2004), all statistical inference is grounded in the Gaussian distribution, supported by the central limit theorem for kernel density estimators. Furthermore, ${\hat{q}}_{T}$ represents the critical value derived from the Gaussian distribution in Section 3.3 of Godtliebsen *et al.* (2002). SSSHiC provides a carefully calibrated multiple comparisons approach to all of these hypotheses, which is based on the concept of the number of independent blocks, that was developed in the context of SiZer by Chaudhuri and Marron (1999).

### 2.4 Definition of cell-type-specific loops

Peaks in the smooths ${\hat{s}}_{h}(i,j)$ often result in statistically significant curvatures at multiple neighboring pixels. Hence, loops are determined by a set of neighboring significant pixels. These sets are defined as clusters of vertically, horizontally or diagonally adjacent pixels.

**Figure 2.**
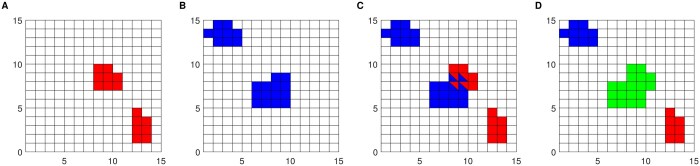
Example of loop clusters from neuron and glia shown in A and B, respectively. C shows the union with some intersections and D illustrates the combined cluster.

Figure 2A shows an example comparing the loop clusters from both neuron and glia. When a neuron cluster and a glia cluster have overlapping pixels, we take the union set of both clusters and let the union become the cluster for each cell type. Panel A shows an example of neuron clusters in red. Panel B shows the same genomic block with significant glia pixels shown in blue. Panel C shows pixels that are significant in either the neuron or the glia analysis, with overlapping pixels colored both red and blue. Next, we color the shared clusters green in D. The resulting graphic highlights individual neuron clusters (neuron specific loops) red, the individual glia clusters (glia specific loops) blue and shared clusters (loops detected in both cell types) green.

## 3 Results

The first part of our results is in Section 3.1 about the fine tuning of SSSHiC to focus on biologically relevant parameters. These are used to find statistically significant loops whose genome ontology is studied in Section 3.2.

### 3.1 Curvature SSS tuning

As in Section 2.1, we applied SSSHiC to sliding windows of size d=1000 for all chromosomes of each cell type, using a window step of d/2=500. In the analysis, we explored different smoothing parameter values, in particular, we considered SSSHiC bandwidth values of $h\in \{2,2^{1.25},2^{1.5},2^{1.75},2^{2},2^{2.25},2^{2.5}\}$. We further considered c=1 to 7 indexing the number of diagonal and off-diagonal bands to be removed. The statistical significance level is always taken as α=0.05.

**Figure 3.**
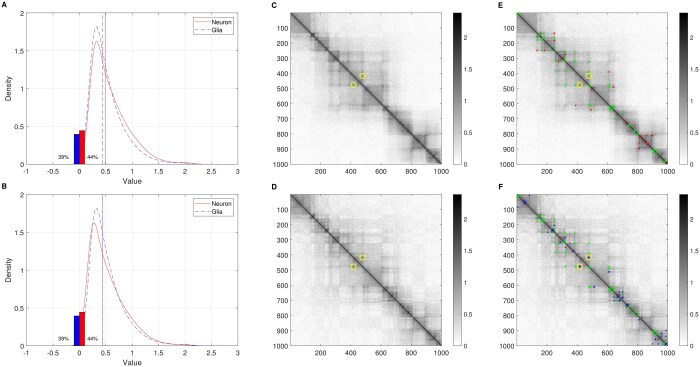
The left panels are density plots for the data before (A) and after (B) median value translation. The bars show the percentages of 0 s and the curves are density estimates of the nonzero values. The middle panel shows pre-processed data in the genomic location of chr5: 125 010 000–135 000 000 for neuron (C) and glia (D). E and F present the significant loops selected by SSSHiC as dots for neuron specific, glia specific, and shared clusters, respectively. The circles highlight a glia-specific loop which is not neuronal for further discussion.

Figure 3 shows an illustrative example of SSSHiC, whose location is chr5: 125 010 000–135 000 000, bandwidth is $h=2^{1.75}$ with c=6, chosen using considerations as follows. The left panels illustrate the median matching preprocessing described in Section 2.2 using kernel density estimates. As in Section 2.2, Figure 3A compares the neuron (red) and glia (blue) pixel log intensity distributions. Note different medians are indicated by the colored vertical lines. Panel B shows the result of the median matching. The resulting neuronal distribution now gives a better fit to the glial distribution. Panels C and D show the median-adjusted heatmaps for neuron (top) and glia (bottom).

In Panels E and F, we first utilized SSSHiC to select significant loops as described in Section 2.3. Next, the significant pixels were clustered into cell-type-specific loops (colored red for neuron, blue for glia) and shared loops (green) as illustrated in Figure 2 of Section 2.4. For example, the blue cluster highlighted by the yellow circle in Panel F appears to be a strong loop at the corresponding position in Panel D. That loop is not significant in Panel E which is sensible because it is not apparent in C.

We next select the parameters, *c* and *h*, of SSSHiC with the multiple goals of (a) maximizing the total number of genes anchored by the discovered loops, (b) identifying cell-type-specific loops containing known cellular marker genes and (c) detecting most loops previously discovered by another loop calling method Mustache.

**Figure 4.**
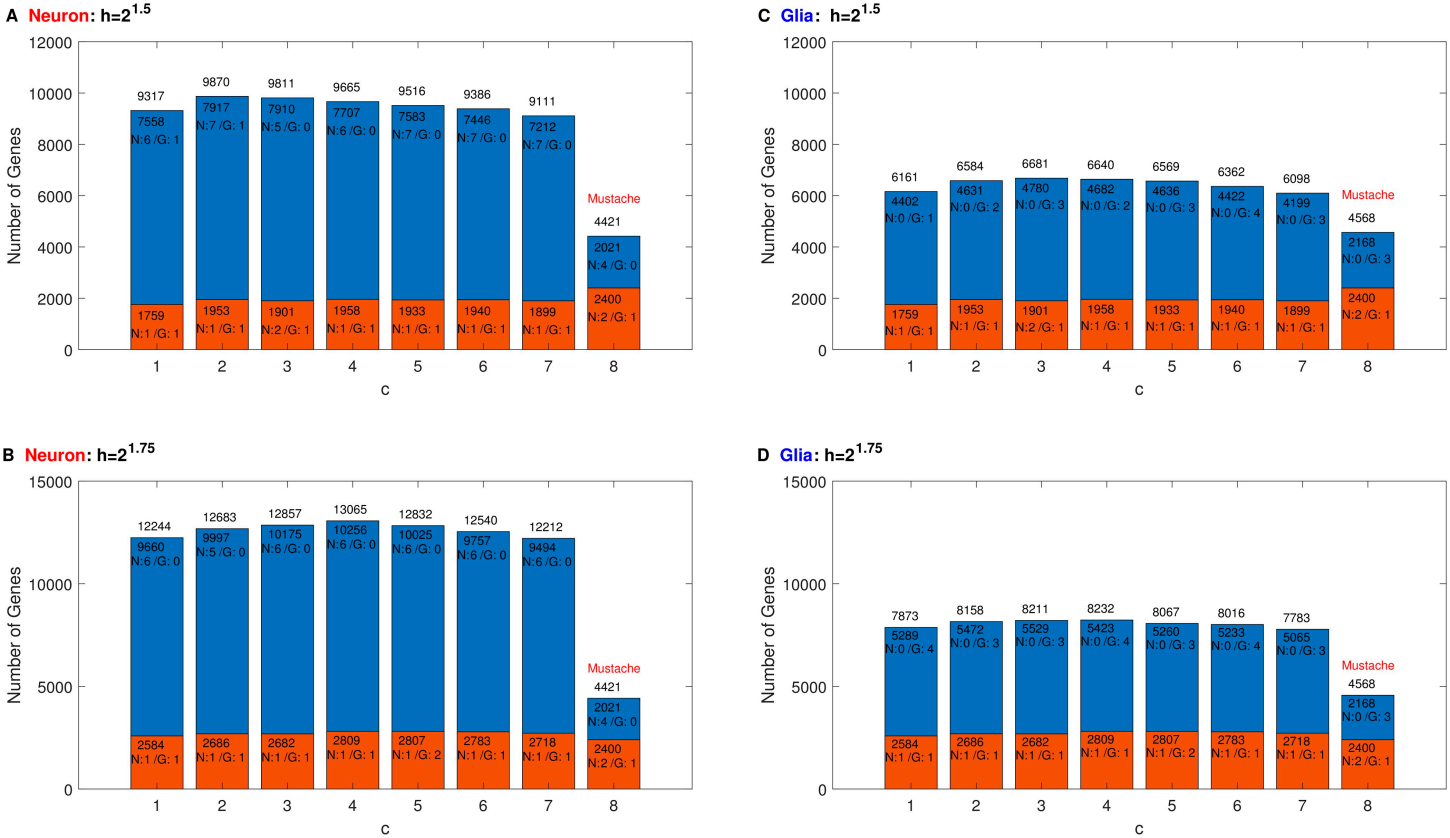
The left panels (A and B) show the selected gene counts from the neuronal contact matrix and the right panels (C and D) show the corresponding glial counts. (A and C) are based on bandwidth $h=2^{1.5}$ while (B and D) use $h=2^{1.75}$. The horizontal axis represents the diagonal lines removed, indexed by c=1 to 7 (the last bar is the result for Mustache as a comparison). Bar heights (with numerical counts shown above each bar) are the numbers of gene promoters located within neuronal or glial loops. The numbers of marker genes in Table 1 detected in cell type specific and shared loops appear (as numbers following N for neuronal and G for glial) at the top of the upper and lower bars, respectively.

For each choice of *c* and *h*, we identified both neuronal and glial-specific loops over the whole genome and identified the promoters located in the discovered loops. For (a), Figure 4 summarizes the result for two chosen bandwidths and a range of *c* values. In the top panels, we study the bandwidth $h=2^{1.5}$, with $h=2^{1.75}$ in the lower panels. The number on top of each bar shows the total number of genes located within significant loops detected for that *c*. These tend to be larger for larger bandwidths. Within each bandwidth, the counts of discovered genes did not vary much for different *c*s. We then stratified these significant loops based on their cell type specificity. Each bar is broken into an orange bar (count near the top) indicating the number of gene promoters located in shared loops and a blue bar (count near the top) showing the number of cell-type-specific promoters. As a comparison, we add a bar showing the corresponding Mustache results on the right in each panel.

**Figure 5.**
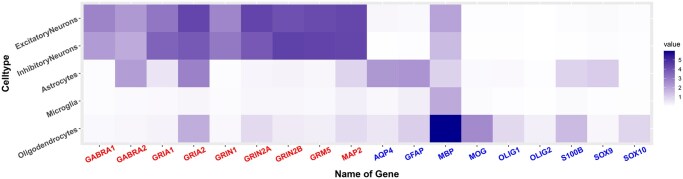
Heatmap of average expression by cell type for each specific gene from Table 1.

For (b), we consider loop detection in nine neuronal and nine glial *marker genes*, given in Table 1. Justification of these gene choices as representatives of neuronal and glial cells, respectively, is given in Figure 5 which shows average gene expression for these genes in primary brain cell types. Furthermore, we aim to include only marker genes of the given cell type while excluding marker genes of the other cell type.

**Table 1.** Target genes’ list for neuron and glia, which includes nine specific genes for neuron and nine for glia

Cell type	Target genes				
Neuron	GABRA1	GABRA2	GRIA1	GRIA2	GRIN1
	GRIN2A	GRIN2B	GRM5	MAP2	
Glia	AQP4	GFAP	MBP	MOG	OLIG1
	OLIG2	S100B	SOX9	SOX10	

We consider the number of correctly located Table 1 marker genes in each case. These are indicated by the numbers in N/G appearing near the top of the blue bar (that cell type only), and orange bar (both cell types). The parameter set $c=6,h=2^{1.5}$ has found seven neuronal marker genes, four glial marker genes, and 0 wrong (N in G or G in N) while $c=1,h=2^{1.75}$, $c=4,h=2^{1.75}$, and $c=6,h=2^{1.75}$ all have found six neuronal marker genes, four glial marker genes, and 0 wrong (N in G or G in N), which are the best performances over all parameter choices.

To compare on the basis of criterion (c), the set of loops for each parameter pair of the top four candidates found by (b) also found by Mustache are counted in Table 2. Among the four top candidates, $c=6,h=2^{1.5}$ performs best with respect to criteria (b) who has found 1 more neuronal marker gene than the other three candidates. However, $c=6,h=2^{1.75}$ has found the best combination of most genes and most overlapping loops with Mustache for both types of cells. Specifically, $c=6,h=2^{1.75}$ has detected 3000 more genes in neuron and 1500 more genes in glia (criterion (a)) and about 1000 more overlaps with Mustache for both types (criterion (c)) than $c=6,h=2^{1.5}$. Overall, we recommend $c=6,h=2^{1.75}$ as optimal with respect to the three criteria (a), (b), and (c).

**Table 2.** Overlapping loops between SSS and Mustache with respect to bandwidth *h* and *c* of the top four candidates for neuron (compared to 9293 loops found by Mustache) and glia (8991 found by Mustache)^a^

Top four candidates			
h	c	Neuron (%)	Glia (%)
$2^{1.5}$	6	4204 (45.2%)	3261 (36.2%)
$2^{1.75}$	1	4649 (50.0%)	3512 (39.0%)
$2^{1.75}$	4	5054 (54.4%)	3976 (44.2%)
$2^{1.75}$	6	5253 (56.5%)	4153 (46.2%)

a The percentages are of the loops which are also found by Mustache.

### 3.2 Characterization of cell-type-specific loops

**Figure 6.**
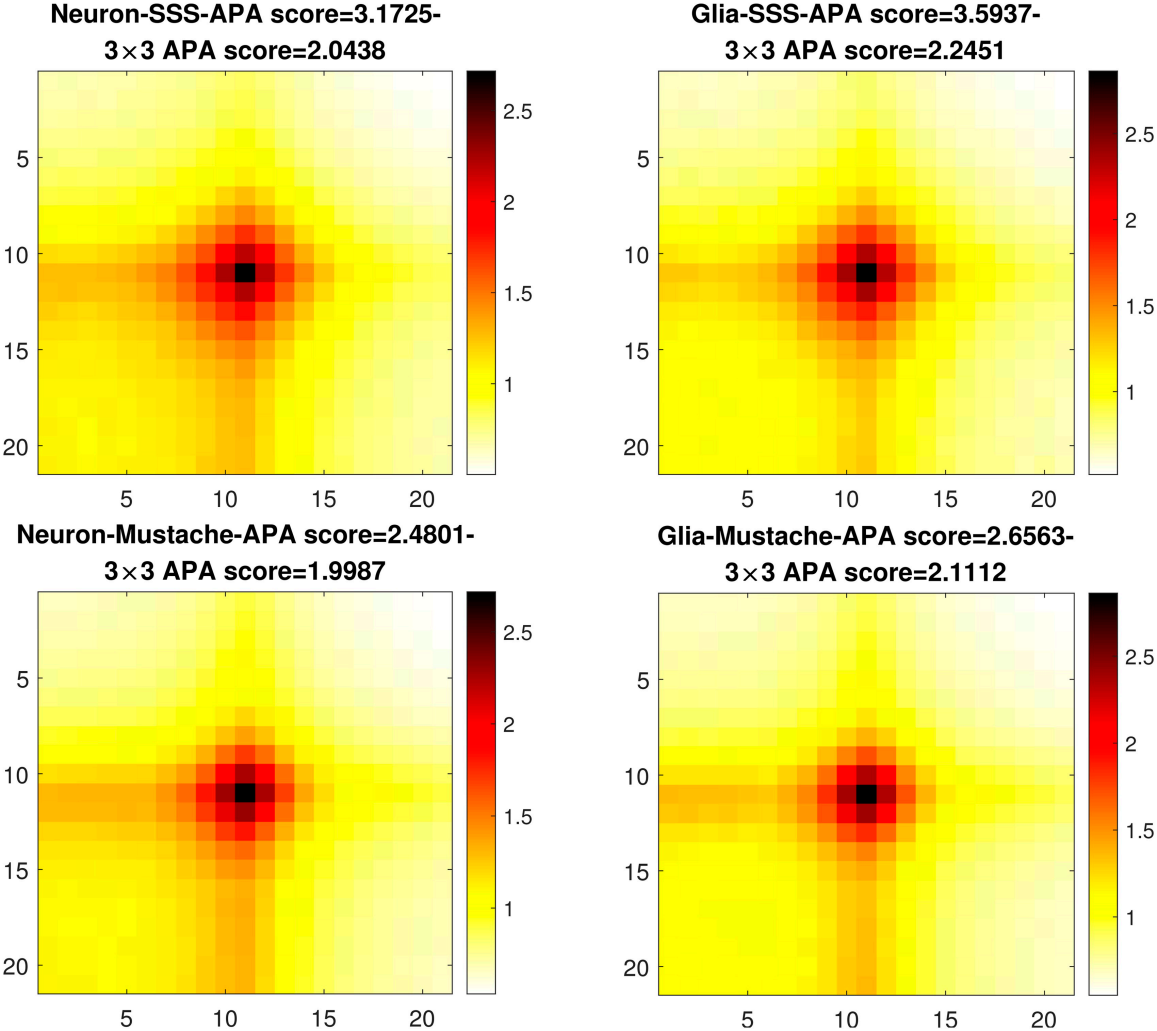
APA plots for SSSHiC with $h=2^{1.75},c=6$ (top row) and Mustache (bottom row) loops in neuron (left column) and glia (right column). The Normalized APA score calculated and 3 × 3 APA score are reported above each plot.

To further evaluate the loops identified by SSSHiC, using our recommended parameters, we assessed the support for each set of loops from the Hi-C data through the use of aggregate peak analysis (APA) (Rao *et al.* 2014). To minimize the impact of distance-related biases, we focused our analysis on loop calls with loop loci separated by more than a minimum threshold of t=300 kb. We employed Normalized APA, which means each submatrix is first normalized before being added to the aggregate matrix. This normalization involves dividing each element by the submatrix’s mean value. The ultimate Normalized APA matrix represents the mean of all these individual submatrices. From Figure 6, loops identified by SSSHiC exhibited more concentrated central enrichment, resulting in higher APA scores in comparison to Mustache. The 3 × 3 APA scores, which measure the mean intensity in the 3 × 3 region around the loop centre, were also higher for SSSHiC than for Mustache.

**Figure 7.**
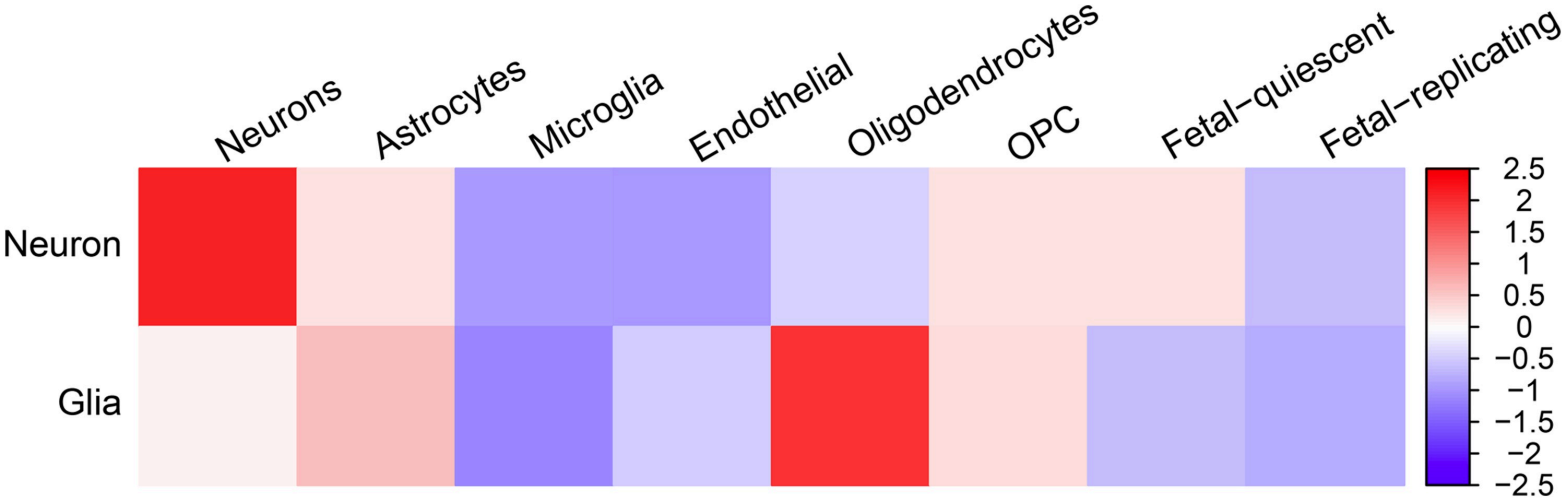
Cell-type expression levels for the cell-type-specific loops from SSSHiC with $h=2^{1.75}$ and c=6 of neuron (top row) and glia (bottom row).

Figure 7 illustrates the expression levels of the genes in Figure 4 in various cell types with our recommended parameters of $h=2^{1.75}$ and c=6, specifically neurons and glia, as indicated by the top and bottom rows, respectively. The heatmap provides a visual comparison across several different cell types and shows that neuron-related genes are significantly expressed in neuronal-specific loops and oligodendrocytes also highly expressed in glial-specific loops. The stark contrast in expression levels between neuron and glial categories provides another validation of these selected loops.

Since loop anchors detected by SSSHiC were enriched for gene promoters, we next assessed their enrichment for enhancers by analyzing the proportion of detected loops that include enhancer elements in their anchors (Hu *et al.* 2021). As shown in Supplementary Table S1, a higher percentage of SSSHiC-detected loops contained enhancers in their anchors compared to Mustache. These results suggest that SSSHiC can capture chromatin loops that are involved in gene regulatory function.

To further assess the effectiveness of SSSHiC, we compared loop calls made by SSSHiC and Mustache with publicly available HiChIP data. In this analysis, we calculated the number of HiChIP loops identified by both SSSHiC and Mustache. As shown in Supplementary Figure S1, the *y*-axis represents the proportion of HiChIP loops that are also detected by either SSSHiC or Mustache. The *x*-axis, labeled ‘Detection Threshold’, indicates the maximum distance within which loops detected by the two algorithms are considered to overlap (in units of 10 kb). As expected, the fraction of overlapping loops increases as the detection threshold grows. We found that both methods effectively recovered loops detected by HiChIP. These results highlight that SSSHiC is comparable to Mustache in detecting loops identified by an orthogonal chromatin capture technology (Ardakany *et al.* 2020).

The distribution of detected loop sizes is presented in Supplementary Figure S2 and Table S2. The histogram in Supplementary Figure S2 illustrates a high density of smaller loops in both neuron and glia cells, with loop sizes predominantly clustering below 500 kb. However, the presence of larger loops, extending up to 3000 kb, is also evident, indicating a substantial range in discovered loop sizes. Supplementary Table S2 further quantifies this distribution, providing specific quantile values that highlight the variability in loop sizes across cell types. This range, from small to large loops, suggests that SSSHiC captures interactions with both close and distant genomic contacts, reflecting diverse regulatory architectures.

## 4 Discussion

In this paper, we proposed SSSHiC, a novel technology to identify chromatin loops from Hi-C data. SSSHiCis a scale space method which can adapt to various levels of resolution through a bandwidth parameter. We applied SSSHiCto two Hi-C datasets acquired from two different brain cell types (neuron and glia) (Hu *et al.* 2021). By identifying loops and carefully defining their cell type specific nature, SSSHiC helps understand chromatin architectural differences and similarities between these two different cell types. In particular, chromatin structure has been shown to play an important role in gene regulation. Given the extensive difference in gene regulation in different cell types, it is expected that chromatin structure in different cell types should reflect such cell type specificity. However, many existing loop callers, such as Mustache, traditionally identified structural loops and failed to capture cell-type-specific chromatin structure which is represented as enhancer–promoter interactions. Compared with Mustache, SSSHiC has detected more loops that are anchored to cell-type-specific marker genes, demonstrating its ability to capture cell-type-specific chromatin structure.

To facilitate the identification of cell-type-specific loops, SSSHiC employs a three-step process:

1. Preprocessing Hi-C data to normalize read depth and remove diagonal elements.
2. Detecting statistically significant loop regions using a curvature-based inference.
3. Classifying loops as shared or cell type specific by comparing clusters of significant pixels across cell types.

This systematic approach enhances the ability of SSSHiC to capture biologically meaningful differences in chromatin architecture.

A key advantage of SSSHiC is that it represents loops as clusters of pixels rather than a single pixel. Existing tools often represent loops as single pixels in the Hi-C contact map. While effective in certain contexts, this approach can limit their ability to accurately capture cell-type-specific interactions. Representing a loop as a single pixel makes it challenging to identify shared loops across cell types, as the overlap between single-pixel loops is minimal, especially when considering biological variability and data noise. Consequently, defining shared loops based on a single pixel requires the introduction of distance thresholds, which may lead to inconsistent or arbitrary overlap definitions and require significant validation. In contrast, SSSHiC represents loops as clusters of pixels rather than single points. This clustering approach allows for a more robust and flexible definition of loops, making it easier to identify and compare shared loops across different cell types. By defining loops as multi-pixel clusters, SSSHiC can account for minor positional shifts that may arise due to biological differences between cell types, experimental noise, or variations in Hi-C resolution. This approach provides a clearer and more consistent basis for identifying cell-type-specific loops, as shared loops can be more reliably defined by the intersection of loop clusters.

Another important contribution of this paper is the invention of novel diagnostic graphics to aid in tuning parameter selection. In particular, simultaneous visualization of the number of loops as well as number of marker genes discovered enables understanding of the trade-offs between our multiple criteria, which guides the selections of the bandwidth (*h*) and diagonal removal (*c*) parameters.

While we are using SSSHiC only for loop detection in this paper, the same methodology can be used for other types of architectural unit detection, such as stripes (Kraft *et al.* 2019, Yoon *et al.* 2022). Detection of stripes requires only shifting the SSSHiC hypothesis from peaks to ridges.

Furthermore, an important statistical issue is that SSSHiC is designed for exploratory data analysis, not confirmatory analysis, in the sense described in Chapter 4 of Marron and Dryden (2021). In particular, it is aimed at suggesting promising avenues of further scientific exploration, as opposed to providing evidence that is sufficiently reliable for say use in a clinical context. When the latter level of certainty is needed, confirmatory analysis such as validation in an independent data set, or at least some type of cross-validation, is essential.

In summary, we found SSSHiC was effective in several aspects, (i) it is applicable to Hi-C data at any resolution because of its scale space view; (ii) relative to other loop callers, such as Mustache, it tends to detect more loops involved in gene regulation that are anchored to gene promoters; and (iii) it defines loops as clusters of pixels rather than the more conventional single pixel for each loop, which reduces the impact of natural variation. Therefore, SSSHiC provides a new, useful, and practical tool to study 3D chromatin structure.

## Supplementary Material

btaf026_Supplementary_Data

## Data Availability

The Hi-C data used in this study can be accessed through the PsychENCODE Knowledge Portal at https://www.synapse.org/#!Synapse:syn21760712.
