# Supplementary material for: Significance in scale space for Hi-C data
Source: Bioinformatics. 2025 Feb 27;41(3):btaf026. doi: 10.1093/bioinformatics/btaf026 (PMC11879645; doi:10.1093/bioinformatics/btaf026)
Supplement: btaf026_Supplementary_Data [file btaf026_supplementary_data.zip › 4da8c_SM_HiC_202408.pdf]

Supplementary Material for Significance in Scale Space for Hi-C Data

Rui Liu *[1], Zhengwu Zhang[1], Hyejung Won[2], and J. S. Marron[1]

[1]Department of Statistics and Operations Research, The University of North Carolina at Chapel Hill
[2]Department of Genetics, The University of North Carolina at Chapel Hill

*correspondingauthor: jerryliu@unc.edu

Supplementary Figures

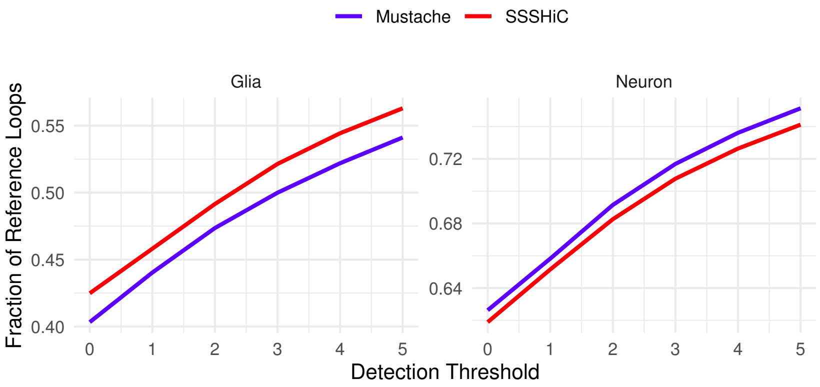

Figure S1: Comparison of loop detection performance between SSSHiC and Mustache across different thresholds for Neuron and Glia cell types, based on overlap with HiChIP data. The x-axis represents threshold values (in units of 10 kb), and the y-axis shows the percentage of detected loops that overlap with HiChIP interactions. Each panel displays results for one cell type: Glia on the left and Neuron on the right.

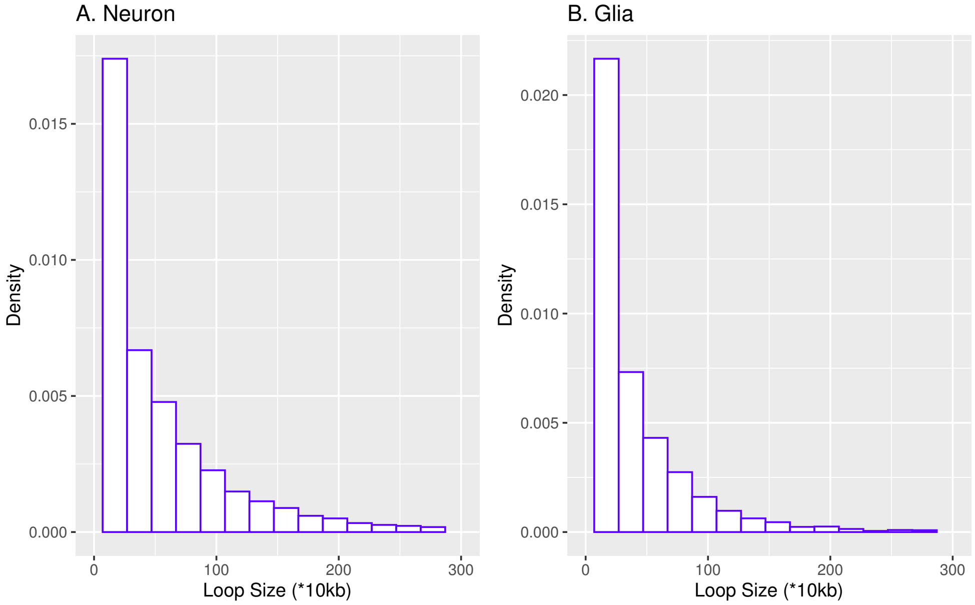

Figure S2: Histogram of loop size distributions in Neuron (A) and Glia (B) cells. Loop sizes are measured in units of 10 kb, indicating the genomic span of each detected loop.

Supplementary Tables

Cell Type	Methods	
	SSSHiC	Mustache
Neuron	83.96%	76.76%
Glia	80.72%	74.35%

Table S1: Percentage of loops with enhancer elements in their anchors detected by SSSHiC and Mustache in neurons and glia.

Cell Type	Quantiles						
	1%	5%	25%	50%	75%	95%	99%
Neuron	7	7	8	22	64	178	320
Glia	7	7	8	14	45	124	255

Table S2: Quantile values of loop size distribution of both Neuron and Glia (loop sizes are measured in units of 10 kb).
